# Marine diversity in the biosphere reserve of the most oceanic island in the Gulf of California: San Pedro Mártir

**DOI:** 10.3897/zookeys.1062.67964

**Published:** 2021-10-15

**Authors:** Imelda G. Amador-Castro, Francisco J. Fernández-Rivera Melo, Jorge Torre

**Affiliations:** 1 Comunidad y Biodiversidad, A.C., Calle Isla del Peruano No. 215, Colonia Lomas de Miramar, C.P. 85448, Guaymas, Sonora, Mexico Comunidad y Biodiversidad Guaymas Mexico

**Keywords:** Diversity, ecological indicator, ichthyofauna, invertebrate, Midriff Islands Region, natural protected area, systematic list

## Abstract

San Pedro Mártir island is of high biological, ecological, and fishery importance and was declared a biosphere reserve in 2002. This island is the most oceanic in the Gulf of California, and information on its rocky reefs is scarce. The present study aimed to generate the first list of conspicuous invertebrate and fish species based on *in situ* observations and to examine the community structure of the shallow rocky reefs of the reserve. In addition, we estimated the ecological indicators of richness, abundance, Shannon diversity, and Pielou evenness to evaluate the conservation status of the biosphere reserve. Data were collected annually from 2007 to 2017 through 2,192 underwater SCUBA transects. A total of 35 species of invertebrates and 73 species of fish were recorded. Most of the species are widely distributed along the eastern Pacific. Overall, 64% of the species found in this study are commercially important, and 11 species have been listed as protected. The abundance of commercially important invertebrate species (i.e., the sea cucumber *Isostichopusfuscus* and the spiny oyster *Spondyluslimbatus*) is decreasing, while commercially important fish species have maintained their abundance with periods of increase. The ecological indicators and the abundance and size of the commercial species indicate that the reserve is in good condition while meeting its conservation objectives.

## Introduction

**Figure 1.**
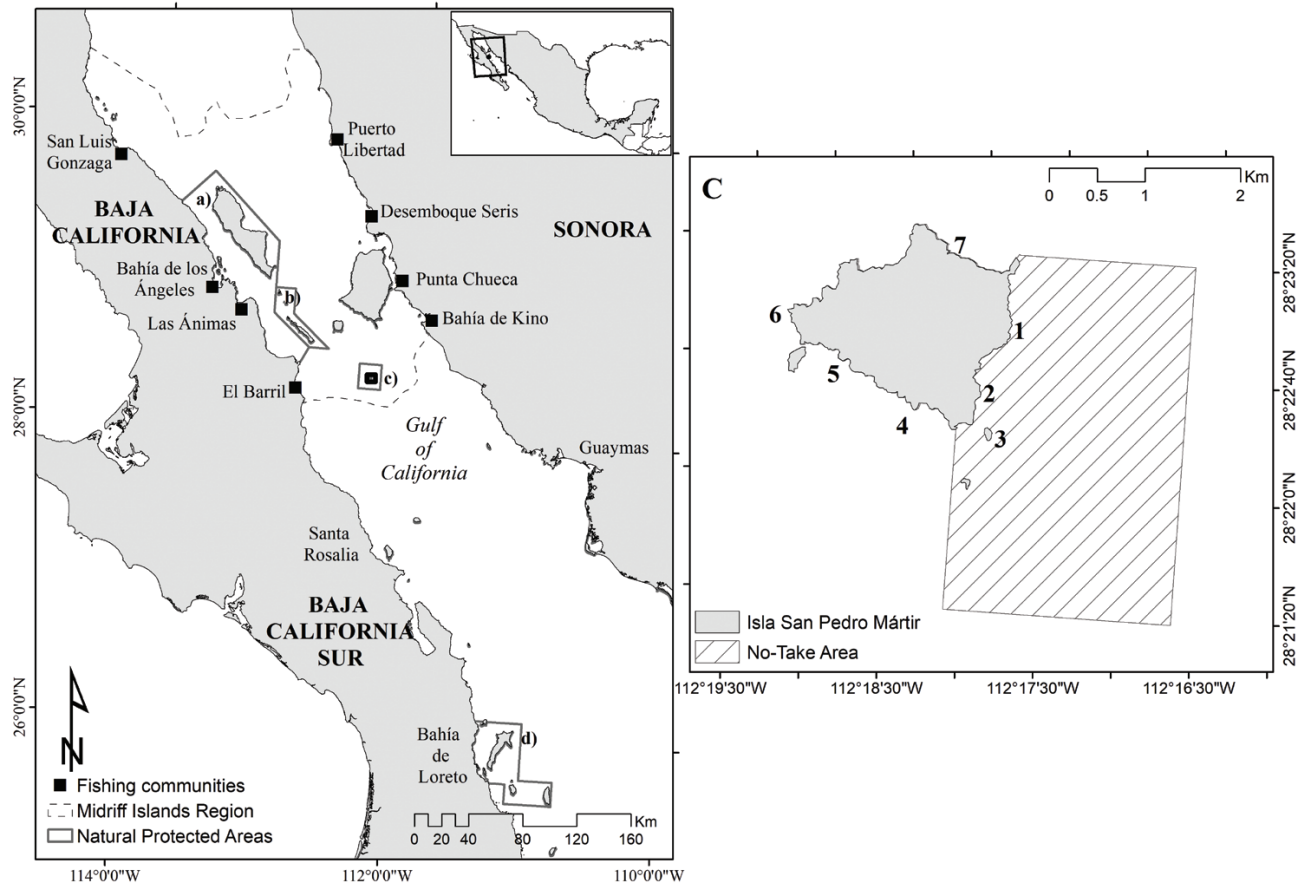
Left panel: Map of the Gulf of California and surrounding areas. The Midriff Islands region (MIR, dotted line) includes the **a** Bahía de los Ángeles, Canales de Ballenas y Salsipuedes Biosphere Reserve; **b** Archipiélago de San Lorenzo National Park; and **c** island San Pedro Mártir Biosphere Reserve (ISPMBR), whereas **d** Bahía de Loreto National Park is located to the south of the MIR. Right panel: The limits of the ISPMBR, including Island San Pedro Mártir (shaded region) and the monitoring sites (**1**–**6**) of this study. **1** Punta Rabijunco **2** Cueva de la Reserva and **3** Los Morritos are located within the core, no-take zone (dashed region) of the ISPMBR, whereas **4** Cueva del Biólogo **5** Barra Baya **6** La Ventana and **7** Arroyo del Cartelón are located within the buffer zone.

The Midriff Islands Region (MIR), which is located near the central portion of the Gulf of California, is an archipelago comprising 45 islands and islets with high biodiversity. Due to this diversity, the MIR is important for conservation, and three natural protected areas have been decreed within its borders: the Archipiélago de San Lorenzo National Park; Bahía de los Ángeles, Canales de Ballenas y Salsipuedes Biosphere Reserve; and Island San Pedro Mártir Biosphere Reserve (ISPMBR; Fig. 1). The coastal rocky reefs of the MIR cover most of its coastlines, and eight fishing communities (Bahía de los Angeles, Bahía de Kino, El Desemboque de los Seris, El Barril, Las Animas, Puerto Libertad, Punta Chueca, and San Luis Gonzaga) depend on the resources found within (Moreno-Báez et al. 2012; Álvarez-Romero et al. 2018).

A large number of studies of ecological indicators and inventories of invertebrate and fish species of the rocky reefs of the Gulf of California have been published, most of which have been conducted in natural protected areas (e.g., Holguín Quiñoez et al. 2000; Villarreal-Cavazos et al. 2000; Brusca et al. 2005; Rodríguez-Romero et al. 2005; Mascareño et al. 2011; Del Moral-Flores et al. 2013; Ayala-Bocos et al. 2018; Fernández-Rivera Melo et al. 2018). In the specific case of the ISPMBR, which contains the most oceanic island in the Gulf of California that is separated from both coasts by an average of 64 km, there is little information available on coastal reef-associated marine fauna. The few studies that have been carried out on the island have focused on generating taxonomic lists with information obtained from the literature, museum inventories, and sparse *in situ* observations (Thomson et al. 2000; Thomson and Guilligan 2002; CONANP 2011; Del Moral-Flores et al. 2013).

Despite the various studies that have been conducted in the MIR, there are currently multiple ecological and biogeographic information gaps and uncertainty regarding the degree to which the conservation objectives of the ISPMBR have been met. As such, the present study aimed to generate a list of conspicuous invertebrate and fish species based on *in situ* observations, in addition to evaluating the community structure and main species (e.g., commercial, endangered, threatened, or protected) present in the shallow rocky reefs (< 20 m depth) of the ISPMBR from 2007 to 2017.

## Materials and methods

### Study area

San Pedro Mártir island is located near the southern border of the MIR, and its polygon (28°18'00"N, 112°13'30"W and 28°28'00"N, 112°23'30"W) is situated between two regions, the northern Gulf of California and central Gulf of California, which have distinct oceanographic (i.e., physical and chemical) characteristics and marine fauna (i.e., invertebrate and fish species; Walter 1960; Brusca et al. 2005; Fernández-Rivera Melo et al. 2018; Fig. 1).

### Sampling and data analysis

From 2007 to 2017, the rocky reefs of three sites within the core zone (Punta Rabijunco, Cueva de la Reserva, and Los Morritos) and four sites within the buffer zone (Arroyo del Cartelón, Cueva del Biólogo, Barra Baya, and La Ventana) of the ISPMBR were monitored (Fig. 1).

Visual surveys were conducted following the underwater monitoring protocols for kelp forests of the Partnership for Interdisciplinary Studies of Coastal Oceans (PISCO 2016). This methodology consists of carrying out band transects, in which macroinvertebrates and conspicuous fish species are identified and counted. Invertebrates were surveyed with band transects measuring 30 m × 2 m (length × width; area of 60 m^2^) at depths of 2, 12, and 18 m. Fish species were surveyed at depths of 5, 10, 15, and 20 m with band transects measuring 30 m × 2 m (length × width; area of 60 m^2^), and fish sizes were estimated (Hernández-Velasco et al. 2018). The entire water column from 2 m above the bottom to the surface was included in each fish transect.

The invertebrate species were classified based on the information available in the World Register of Marine Species (WoRMS 2019), and data on their distributions were obtained from Palomares and Pauly (2020). Fish species were classified using the Catalog of Fishes of the California Academy of Sciences (Eschmeyer et al. 2021), and their distributions were determined based on those from Froese and Pauly (2019). In addition, the threat categories of at-risk species were also determined using the Red List of the International Union for Conservation of Nature (IUCN) and the Official Mexican Standard of at-risk species of flora and fauna NOM-059-SEMARNAT-2010. Lastly, commercially important species were identified in the ISPMBR.

The data collected during sampling were used to estimate the ecological indicators of abundance, richness (S), diversity (i.e., Shannon-Wiener diversity index; ln; H’), and evenness (Pielou evenness index; J’), which are the most commonly used indices that have been used to evaluate community structure in the Gulf of California. These indices were used to compare community structure between the buffer and core zones of the ISPMBR and among monitoring years. Estimations of the ecological indicators were carried out with PAST v. 4.06 (Hammer 2001), whereas statistical analyses were carried out in JAMOVI v. 1.6 (The jamovi project). When no significant differences were found between the buffer zone and core zones with regard to the three ecological indicators (Suppl. material 1: Table S1), the information is presented only according to monitoring year. In addition, we estimated a series of alpha diversity metrics, including rank abundance curves given that they are complementary to multivariate methods and detail species-level community changes (species richness, species evenness, species gain and loss, and curve changes over time), using the R package ‘codyn’ (Avolio et al. 2019).

## Results

During the eleven years of this study, a total of 2,192 transects were surveyed (730 and 1,462 invertebrate and fish transects, respectively). Overall, 31,766 invertebrate individuals belonging to 35 species, 20 genera, and 27 families were recorded. The Muricidae family was the most represented in this study with three species, while 19 families were represented by one species (Table 1). A total of 167,242 fish individuals belonging to 73 species, 49 genera, and 27 families were identified. The Serranidae and Labridae families were the most highly represented with 11 species and 10 species, respectively (Table 2).

**Table 1.** Systematic list of invertebrates in the Island San Pedro Mártir Biosphere Reserve (ISPMBR) in the Gulf of California, Mexico. The biogeographic region of each species is shown. Abbreviations: EP = eastern Pacific; EP + WCA = eastern Pacific + western central Atlantic; EP + NEA = eastern Pacific + northeastern Atlantic; EP + SA = eastern Pacific + southern Atlantic; ECP = eastern central Pacific; SP = southeastern Pacific; CA = Central America; IP = Indo-Pacific; IP + WCA = Indo-Pacific + western central Atlantic; PO = Pacific Ocean; N/A = No information available; IUCN = International Union for Conservation of Nature; NOM-59 = Mexican law for endangered, threatened, or protected species (NOM-059-SEMARNAT-2010).

Taxa		Species	Species at risk or under protection		Biogeographic region	Commercial importance
			IUCN	NOM-059		
**MOLLUSCA**	BIVALVIA					
	**Ostreida**					
	Gryphaeidae	*Hyotissahyotis* (Linnaeus, 1758)	Not evaluated		IP + WCA	
	Margaritidae	*Pinctadamazatlanica* (Hanley, 1856)	Not evaluated	Subject to special protection	EP	•
	Pinnidae	*Pinnarugosa* G. B. Sowerby I, 1835	Not evaluated		EP + NEA	•
	Pteriidae	*Pteriasterna* (Gould, 1851)	Not evaluated		EP	•
	Spondylidae	*Spondyluslimbatus* G. B. Sowerby II, 1847	Not evaluated	Subject to special protection	IP	•
	CEPHALOPODA					
	**Order Octopoda**					
	Octopodidae	*Octopusbimaculatus* Verrill, 1883	Minor concern		PO	•
	GASTROPODA					
	**Littorinimorpha**					
	Strombidae	*Strombusgracilior* G. B. Sowerby I, 1825	Not evaluated		EP	•
		*Titanostrombusgaleatus* (Swainson, 1823)	Not evaluated		EP +SA	
	**Neogastropoda**					
	Columbellidae	*Strombinamaculosa* (G. B. Sowerby I, 1832)	Not evaluated		N/A	
	Fasciolariidae	*Triplofususprinceps* (G. B. Sowerby I, 1825)	Not evaluated		EP	
	Muricidae	*Hexaplexerythrostomus* (Swainson, 1831)	Not evaluated		CA	•
		*Hexaplexnigritus* (Philippi, 1845)	Not evaluated		N/A	•
		*Hexaplexprinceps* (Broderip, 1833)	Not evaluated		SP	•
	**Trochida**					
	Turbinidae	*Turbofluctuosus* W. Wood, 1828	Not evaluated		N/A	
**ECHINODERMATA**	ASTEROIDEA					
	**Forcipulatida**					
	Heliasteridae	*Heliasterkubiniji* Xantus, 1860	Not evaluated		N/A	
	**Spinulosida**					
	Echinasteridae	*Echinastertenuispina* Verrill, 1871	Not evaluated		N/A	
	**Valvatida**					
	Acanthasteridae	*Acanthasterplanci* (Linnaeus, 1758)	Not evaluated		IP	
	Asteropseidae	*Asteropsiscarinifera* (Lamarck, 1816)	Not evaluated		IP	•
	Mithrodiidae	*Mithrodiabradleyi* Verrill, 1867	Not evaluated		N/A	
	Ophidiasteridae	*Phariapyramidata* (Gray, 1840)	Not evaluated		EP	•
		*Phatariaunifascialis* (Gray, 1840)	Not evaluated		EP	•
	Oreasteridae	*Nidorelliaarmata* (Gray, 1840)	Not evaluated		EP + WCA	•
		*Pentacerastercumingi* (Gray, 1840)	Not evaluated		EP	•
**ECHINOIDEA**	**Arbacioida**					
	Arbaciidae	*Arbaciastellata* (Blainville, 1825; Gmelin, 1791)	Not evaluated		EP	•
	**Camarodonta**					
	Echinometridae	*Echinometravanbrunti* A. Agassiz, 1863	Not evaluated		EP	
	Toxopneustidae	*Toxopneustesroseus* (A. Agassiz, 1863)	Not evaluated		EP	
		*Tripneustesdepressus* A. Agassiz, 1863	Not evaluated		EP	
	**Cidaroida**					
	Cidaridae	*Eucidaristhouarsii* (L. Agassiz & Desor, 1846)	Not evaluated		EP	•
	**Diadematoida**					
	Diadematidae	*Centrostephanuscoronatus* (Verrill, 1867)	Not evaluated		EP	•
**ECHINOIDEA**	Diadematidae	*Diademamexicanum* A. Agassiz, 1863	Not evaluated		EP	
	**HOLOTHUROIDEA**					
	**Synallactida**					
	Stichopodidae	*Isostichopusfuscus* (Ludwig, 1875)	In danger of extinction	Subject to special protection	EP	•
**CNIDARIA**	**ANTHOZOA**					
	**Antipatharia**					
	Antipathidae	*Antipathesgalapagensis* Deichmann, 1941	Not evaluated		EP	
**ARTHROPODA**	**MALACOSTRACA**					
	**Decapoda**					
	Inachoididae	*Stenorhynchusdebilis* (Smith, 1871)	Not evaluated		SP	
	Palinuridae	*Panulirusinflatus* (Bouvier, 1895)	Minor concern		ECP	•
		*Panulirusinterruptus* (Randall, 1840)	Minor concern		ECP	•

**Table 2.** Systematic list of fish species in the Island San Pedro Mártir Biosphere Reserve (ISPMBR) in the Gulf of California, Mexico. The biogeographic region of each species is shown, as well as the average, minimum (min), and maximum (max) sizes (cm) based on the survey data. Abbreviations: EP = eastern Pacific; ECP=eastern central Pacific; C = Circumglobal; CT = Circumtropical; CT + EP = Circumtropical+eastern Pacific; IP = Indo-Pacific; IP + EP = Indo-Pacific + eastern Pacific; IUCN = International Union for Conservation of Nature; NOM-59 = Mexican law for endangered, threatened, or protected species (NOM-059-SEMARNAT-2010).

	Taxa		Species	Species at risk or under protection		Biogeographic region	Commercial importance	Average size (min, max)
				IUCN	NOM-059			
	**ELASMOBRANCHII**	**Heterodontiformes**						
		Heterodontidae	*Heterodontusfrancisci* (Girard 1855)	Insufficient data		EP	•	45.00 (30,60)
		**Torpediniformes**						
		Narcinidae	*Diplobatisommata* (Jordan & Gilbert 1890)	Vulnerable		EP	•	20.00 (20,20)
			*Narcineentemedor* Jordan & Starks 1895	Insufficient data		EP	•	18.33 (15,20)
		**Rhinopristiformes**						
		Rhinobatidae	*Pseudobatosproductus* (Ayres 1854)	Near threatened		EP	•	66.67 (20,100)
		**Myliobatiformes**						
		Urotrygonidae	*Urobatisconcentricus* Osburn & Nichols 1916	Insufficient data		ECP	•	29.63 (15,40)
	**ACTINOPTERI**	**Anguilliformes**						
		Muraenidae	*Gymnothoraxcastaneus* (Jordan & Gilbert 1883)	Minor concern		EP		58.64 (30,100)
			*Gymnothoraxdovii* (Günther 1870)	Minor concern		EP		No data
			*Muraenalentiginosa* Jenyns 1842	Minor concern		EP		37.78 (20,60)
		**Blenniiformes**						
		Blenniidae	*Ophioblenniussteindachneri* Jordan & Evermann 1898	Minor concern		EP		9.47 (5,15)
		**Acanthuriformes**						
		Pomacanthidae	*Holacanthusclarionensis* Gilbert 1890	Vulnerable	Subject to special protection	ECP	•	20.00 (20,20)
			*Holacanthuspasser* Valenciennes 1846	Minor concern	Subject to special protection	EP	•	21.59 (3,40)
			*Pomacanthuszonipectus* (Gill 1862)	Minor concern	Subject to special protection	EP	•	29.42 (15,40)
		Chaetodontidae	*Chaetodonhumeralis* Günther 1860	Minor concern		EP	•	14.52 (5,20)
			*Johnrandallianigrirostris* (Gill 1862)	Minor concern		EP		13.56 (3,30)
		Acanthuridae	*Prionuruslaticlavius* Gill 1862	Minor concern		ECP		36.24 (5,60)
		**Tetraodontiformes**						
		Diodontidae	*Diodonholocanthus* Linnaeus 1758	Minor concern		CT	•	24.78 (12,40)
			*Diodonhystrix* Linnaeus 1758	Minor concern		CT+ EP	•	24.63 (12,30)
		Tetraodontidae	*Canthigasterpunctatissima* (Günther 1870)	Minor concern		ECP	•	6.74 (3,10)
			*Sphoeroideslobatus* (Steindachner 1870)	Minor concern		EP		20.00 (20,20)
		Balistidae	*Balistespolylepis* Steindachner 1876	Minor concern		EP	•	27.60 (3,50)
			*Sufflamenverres* (Gilbert & Starks 1904)	Minor concern		EP		23.57 (10,30)
		**Centrarchiformes**						
		Kyphosidae	*Kyphosusvaigiensis* (Quoy & Gaimard 1825)	Minor concern		C		30.00 (25,40)
			*Kyphosusazureus* (Jenkins & Evermann 1889)	Minor concern		ECP		25.00 (20,30)
		Kyphosidae	*Kyphosuselegans* (Peters 1869)	Minor concern		EP		29.17 (25,30)
		Girellidae	*Girellasimplicidens* Osburn & Nichols 1916	Minor concern		ECP	•	27.32 (3,40)
		Cirrhitidae	*Cirrhitichthysoxycephalus* (Bleeker 1855)	Minor concern		IP	•	6.04 (3,10)
			*Cirrhitusrivulatus* Valenciennes 1846	Minor concern		ECP	•	30.08 (10,45)
**ACTINOPTERI**	**Perciformes**	Serranidae	*Alphestesimmaculatus* Breder 1936	Minor concern		EP	•	17.34 (3,30)
			*Cephalopholispanamensis* (Steindachner 1876)	Minor concern		EP	•	22.98 (5,40)
			*Epinephelusanalogus* Gill 1863	Minor concern		EP	•	22.50 (20,25)
			*Epinepheluslabriformis* (Jenyns 1840)	Minor concern		EP	•	24.67 (10,40)
			*Mycteropercajordani* (Jenkins & Evermann 1889)	In danger of extinction		ECP	•	81.56 (20,150)
			*Mycteropercaprionura* Rosenblatt & Zahuranec 1967	Insufficient data		ECP	•	32.12 (10,50)
			*Mycteropercarosacea* (Streets 1877)	Minor concern		ECP	•	30.25 (5,90)
			*Paralabraxauroguttatus* Walford 1936	Insufficient data		ECP	•	30.00 (30,30)
			*Paralabraxmaculatofasciatus* (Steindachner 1868)	Minor concern		ECP	•	22.50 (15,30)
			*Paranthiascolonus* (Valenciennes 1846)	Minor concern		EP		20.78 (3,40)
			*Serranuspsittacinus* Valenciennes 1846	Minor concern		EP		7.83 (3,20)
		Apogonidae	*Apogonpacificus* (Herre 1935)	Minor concern		EP		5 (5,5)
			*A retrosella* (Gill 1862)	Minor concern		ECP		5.13 (3,10)
		Carangidae	*Seriolalalandi* Valenciennes 1833	Minor concern		C	•	67.50 (60,100)
		Lutjanidae	*Hoplopagrusguentherii* Gill 1862	Minor concern		EP	•	33.55 (5,50)
			*Lutjanusargentiventris* (Peters 1869)	Minor concern		EP	•	31.52 (3,50)
			*Lutjanusviridis* (Valenciennes 1846)	Minor concern		EP	•	25.00 (25,25)
		Haemulidae	*Anisotremusdavidsonii* (Steindachner 1876)	Minor concern		ECP	•	40 (40,40)
			*Anisotremusinterruptus* (Gill 1862)	Minor concern		EP	•	31.32 (10,40)
			*Haemulonflaviguttatum* Gill 1862	Minor concern		EP	•	26.25 (20,30)
			*Haemulonmaculicauda* (Gill 1862)	Minor concern		EP	•	23.19 (6,30)
			*Haemulonsexfasciatum* Gill 1862	Minor concern		ECP	•	29.47 (3,45)
		Sparidae	*Calamusbrachysomus* (Lockington 1880)	Minor concern		EP	•	23.49 (15,30)
		Sciaenidae	*Parequesfuscovittatus* (Kendall & Radcliffe 1912)	Minor concern		ECP		16.21 (10,20)
		Mullidae	*Mulloidichthysdentatus* (Gill 1862)	Minor concern		EP		15 (15,15)
		Pomacentridae	*Abudefduftroschelii* (Gill 1862)	Minor concern		EP		12.44 (5,20)
			*Azurinaatrilobata* Gill 1862	Minor concern		EP	•	5.76 (3,15)
			*Chromislimbaughi* Greenfield & Woods 1980	Minor concern	Subject to special protection	ECP	•	4.67 (3,15)
			*Microspathodondorsalis* (Gill 1862)	Minor concern		EP	•	25.00 (20,30)
			*Stegastesacapulcoensis* (Fowler 1944)	Minor concern		EP		7.14 (5,10)
			*Stegastesflavilatus* (Gill 1862)	Minor concern		EP		8.50 (5,10)
			*Stegastesrectifraenum* (Gill 1862)	Minor concern		ECP		6.70 (3,25)
		Labridae	*Bodianusdiplotaenia* (Gill 1862)	Minor concern		EP	•	20.10 (3,50)
			*Halichoereschierchiae* Di Caporiacco 1948	Minor concern		ECP		9.63 (3,20)
			*Halichoeresdispilus* (Günther 1864)	Minor concern		EP		7.96 (3,25)
			*Halichoeresmelanotis* (Gilbert 1890)	Minor concern		ECP		6.29 (3,15)
			*Halichoeresnicholsi* (Jordan & Gilbert 1882)	Minor concern		EP	•	13.13 (3,40)
			*Halichoeresnotospilus* (Günther 1864)	Minor concern		EP		10.17 (3,25)
			*Halichoeressemicinctus* (Ayres 1859)	Minor concern		EP		16.96 (5,35)
			*Semicossyphuspulcher* (Ayres 1854)	Vulnerable		EP	•	38.13 (30,50)
			*Thalassomagrammaticum* Gilbert 1890	Minor concern		EP		7.50 (5,10)
			*Thalassomalucasanum* (Gill 1862)	Minor concern		EP		6.75 (3,20)
		Scaridae	*Nicholsinadenticulata* (Evermann & Radcliffe 1917)	Minor concern		EP	•	19.30 (5,30)
			*Scaruscompressus* (Osburn & Nichols 1916)	Minor concern		EP		32.00 (10,60)
			*Scarusghobban* Forsskål 1775	Minor concern		IP+ EP		27.25 (8,40)
		Scorpaenidae	*Scorpaenamystes* Jordan & Starks 1895	Minor concern		EP		22.28 (3,40)

### Endangered, threatened, and protected (ETP) species

The Red List of the IUCN classified six of the species reported in this study (a sea cucumber, two rays, and three teleosts) in some risk category. The shovelnose guitarfish (*Pseudobatosproductus*) is classified as Near Threatened, whereas the ocellated electric ray (*Diplobatisommata*), clarion angelfish (*Holcanthusclarionensis*), and California sheephead (*Semicossyphuspulcher*) are classified as Vulnerable. The brown sea cucumber (*Isostichopusfuscus*) and gulf grouper (*Mycteropercajordani*) are listed as Endangered (IUCN 2020).

In the Official Mexican Standard NOM-059-SEMARNAT-2010, six species (three invertebrate and four fish species) are listed as in need of special protection: the pearl oyster (*Pinctadamazatlanica*), spiny oyster (*Spondyluslimbatus*), clarion angelfish (*H.clarionensis*), king angelfish (*Holacanthuspasser*), Cortez angelfish (*Pomacanthuszonipectus*), and Limbaugh’s damselfish (*Chromislimbaughi*), while the brown sea cucumber (*I.fuscus*) is listed as endangered.

The abundance of ETP species varied within the ISPMBR during the study period; for example, *H.clarionensis* were only recorded in 2009, and *P.productus* and *D.ommata* were recorded in two years (Table 3). The species that showed a decrease in abundance over the study period were *I.fuscus* (from 0.77 to 0.01 ind./60 m^2^) and *S.limbatus* (from 0.27 to 0 ind./60 m^2^), which are two commercially important species in the region. The most stable populations were those of *H.passer* and *P.zonipectus*, which maintained abundance values ​​between 1.75–2.40 ind./60 m^2^ and 0.03–0.07 ind./ 60 m^2^, respectively (Table 3).

**Table 3.** Average abundance (standard deviation) in 60 m^2^ of endangered, threatened, and protected eastern tropical Pacific species in the Island San Pedro Mártir Biosphere Reserve (ISPMBR) in the Gulf of California, Mexico.

**Invertebrates**	**2007**	**2008**	**2009**	**2010**	**2011**	**2012**	**2013**	**2014**	**2015**	**2016**	**2017**
	n = 30	n = 72	n = 67	n = 72	n =67	n =74	n = 72	n = 79	n = 53	n = 72	n = 72
*Pinctadamazatlanica*	0.10	0.14	0.06	0.08	0.16	0.05	0.10	0.04	0.04	0.03	0.15
	(0.40)	(0.51)	(0.24)	(0.28)	(0.54)	(0.28)	(0.42)	(0.25)	(0.19)	(0.17)	(0.55)
*Spondyluslimbatus*	0.27	0.22	0.04	0.01	0.07	0.07	0.03	0.09	0.02	0.00	0.00
	(0.69)	(0.86)	(0.27)	(0.12)	(0.31)	(0.34)	(0.17)	(0.40)	(0.14)	(0.00)	(0.00)
*Isostichopusfuscus*	0.77	0.64	0.21	0.22	0.27	0.05	0.38	0.05	0.11	0.15	0.01(0.12)
	(1.79)	(1.08)	(0.45)	(0.48)	(0.62)	(0.28)	(0.88)	(0.22)	(0.42)	(0.39)	(0.12)
**Fishes**	**2007**	**2008**	**2009**	**2010**	**2011**	**2012**	**2013**	**2014**	**2015**	**2016**	**2017**
	n = 76	n = 143	n = 144	n = 144	n =141	n =135	n = 144	n = 132	n = 109	n = 144	n = 150
*Diplobatisommata*	0.01	0.00	0.01	0.00	0.00	0.00	0.00	0.00	0.00	0.00	0.00
	(0.11)	(0.00)	(0.12)	(0.00)	(0.00)	(0.00)	(0.00)	(0.00)	(0.00)	(0.00)	(0.00)
*Pseudobatosproductus*	0.03	0.00	0.00	0.00	0.00	0.01	0.00	0.00	0.00	0.00	0.00
	(0.16)	(0.00)	(0.00)	(0.00)	(0.00)	(0.09)	(0.00)	(0.00)	(0.00)	(0.00)	(0.00)
*Mycteropercajordani*	0.01	0.13	0.12	0.19	0.05	0.18	0.01	0.05	0.02	0.00	0.02
	(0.11)	(0.61)	(0.38)	(0.71)	(0.25)	(1.58)	(0.08)	(0.27)	(0.13)	(0.00)	(0.14)
*Chromislimbaughi*	0.08	0.16	0.59	0.24	0.74	0.10	0.04	0.00	0.00	0.06	0.03
	(0.58)	(1.00)	(3.31)	(1.12)	(4.13)	(0.75)	(0.35)	(0.00)	(0.00)	(0.67)	(0.41)
*Holacanthusclarionensis*	0.00	0.00	0.02	0.00	0.00	0.00	0.00	0.00	0.00	0.00	0.00
	(0.00)	(0.00)	(0.25)	(0.00)	(0.00)	(0.00)	(0.00)	(0.00)	(0.00)	(0.00)	(0.00)
*Holacanthuspasser*	4.17	2.40	2.34	2.20	2.63	2.03	2.17	2.49	2.06	2.24	1.75
	(6.56)	(2.79)	(2.34)	(2.39)	(3.06)	(2.65)	(2.26)	(3.15)	(2.06)	(3.93)	(1.70)
*Pomacanthuszonipectus*	0.18	0.03	0.13	0.07	0.04	0.04	0.03	0.07	0.07	0.06	0.07
	(0.86)	(0.22)	(0.68)	(0.37)	(0.20)	(0.23)	(0.16)	(0.31)	(0.35)	(0.34)	(0.34)
*Semicossyphuspulcher*	0.00	0.00	0.00	0.00	0.00	0.00	0.01	0.00	0.00	0.03	0.01
	(0.00)	(0.00)	(0.00)	(0.00)	(0.00)	(0.00)	(0.08)	(0.00)	(0.00)	(0.18)	(0.12)

### Biogeographic affinity

Of the 35 invertebrate species registered in the ISPMBR, 45.71% are distributed in the eastern Pacific, 8.57% are distributed in the Eastern Indo-Pacific, and 5.71% are distributed in the southeastern Pacific and eastern central Pacific. Finally, no information was found on the distributions of 17.14% of the invertebrate species registered in this study.

Of the 73 registered fish species, four species are solely distributed in Mexico (*Parequesfuscovittatus*, *Girellasimplicidens*, *C.limbaughi*, and *Stegastesrectifraenum*), and two of these (*C.limbaughi* and *S.rectifraenum*) are only distributed in the Gulf of California. Most of the fish species (64.38%) are widely distributed in the eastern Pacific, and 27.20% are species with a biogeographic affinity for the central eastern Pacific. A total of 2.74% of the fish species are distributed in the Indo-Pacific, and 1.37% of the fish species showed circumglobal and circumtropical distributions, respectively.

### Richness, abundance, diversity, and evenness

**Figure 2.**
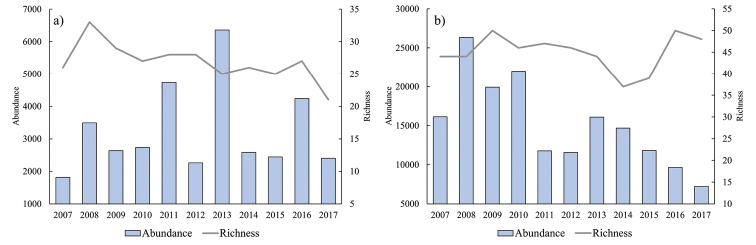
Total abundance and number of **a** invertebrate and **b** fish species observed per year in the Island San Pedro Mártir Biosphere Reserve (ISPMBR) in the Gulf of California, Mexico.

The year with the highest total richness of invertebrates was 2008 (33 species), while the lowest richness value was present in 2017 (21 species). The highest total recorded abundance of invertebrates was 6,359 individuals in 2013. For reef fishes, the years with the highest recorded richness values were 2009 and 2016 with 50 species recorded in each year, while the lowest number of species (37) was recorded in 2014. The highest fish abundance during the study period was 26,332 individuals, which was recorded in 2008 (Fig. 2).

Of the 108 species observed in the ISPMBR, the most abundant invertebrate species were the state pencil urchin (*Eucidaristhouarsii*), blue sea star (*Phatariaunifascialis*), and yellow spotted star (*Phariapyramidata*; Suppl. material 2: Table S2), whereas the most abundant fish species were scissortail damselfish (*Azurinaatrilobata*), Cortez damselfish (*S.rectifraenum*), and Cortez rainbow wrasse (*Thalassomalucasanum*; Suppl. material 3: Table S3). A total of 19 species were recorded (five invertebrates and 14 fish species) with total abundance values of fewer than five individuals during the 10 years of this study (Suppl. material 2: Table S2).

A total of 54 commercially important species (12 invertebrate and 42 fish species) for the communities of the MIR were recorded (Table 1 and Table 2). Among the highly abundant, commercially important species in the ISPMBR were the leopard grouper (*Mycteropercarosacea*) and finescale triggerfish (*Balistespolylepis*; Suppl. material 3: Table S3). The average sizes increased over the study period for *B.polylepis* (from 23.75 cm in 2007 to 28.39 cm in 2017), *M.rosacea* (from 21.86 cm in 2007 to 34.08 cm in 2017), and *Lutjanusargentiventris* (from 20.00 cm in 2007 to 34.00 cm in 2017; Table 4).

**Table 4.** Average size in centimeters (standard deviation) of the main commercially important fish species in the Island San Pedro Mártir Biosphere Reserve (ISPMBR) in the Gulf of California, Mexico. Abbreviations: ND = Not enough data.

Species	2007	2008	2009	2010	2011	2012	2013	2014	2015	2016	2017
	n = 76	n = 143	n = 144	n = 144	n = 141	n = 135	n = 144	n = 132	n = 109	n = 144	n = 150
*Balistespolylepis*	23.75	23.33	26.67	26.92	28.07	27.33	30.12	27.55	27.46	26.57	28.39
	(5.82)	(6.57)	(5.40)	(5.84)	(5.23)	(6.94)	(4.15)	(5.58)	(7.22)	(5.66)	(6.06)
*Mycteropercajordani*	120.00	84.29	85.07	94.62	75.00	82.50	120.00	22.50	47.50	0.00	90.00
	(ND)	(49.95)	(24.28)	(21.06)	(37.28)	(45.00)	(ND)	(5.00)	(31.82)	(ND)	(26.46)
*Mycteropercaprionura*	0.00	38.33	31.32	22.50	25.00	25.00	30.00	32.50	32.50	40.00	45.00
	(ND)	(11.69)	(2.50)	(10.61)	(ND)	(13.23)	(ND)	(2.89)	(3.54)	(ND)	(ND)
*Mycteropercarosacea*	21.86	25.90	25.77	30.56	31.78	32.84	30.69	33.51	35.49	28.61	34.08
	(11.31)	(11.11)	(9.82)	(12.98)	(9.72)	(10.74)	(11.99)	(9.78)	(11.84)	(13.40)	(12.45)
*Lutjanusargentiventris*	20.00	30.00	29.31	31.47	32.50	31.43	31.64	30.60	35.00	30.87	34.00
	(10.00)	(5.53)	(8.21)	(4.0)	(5.34)	(5.73)	(7.69)	(7.94)	(3.97)	(8.48)	(ND)

**Figure 3.**
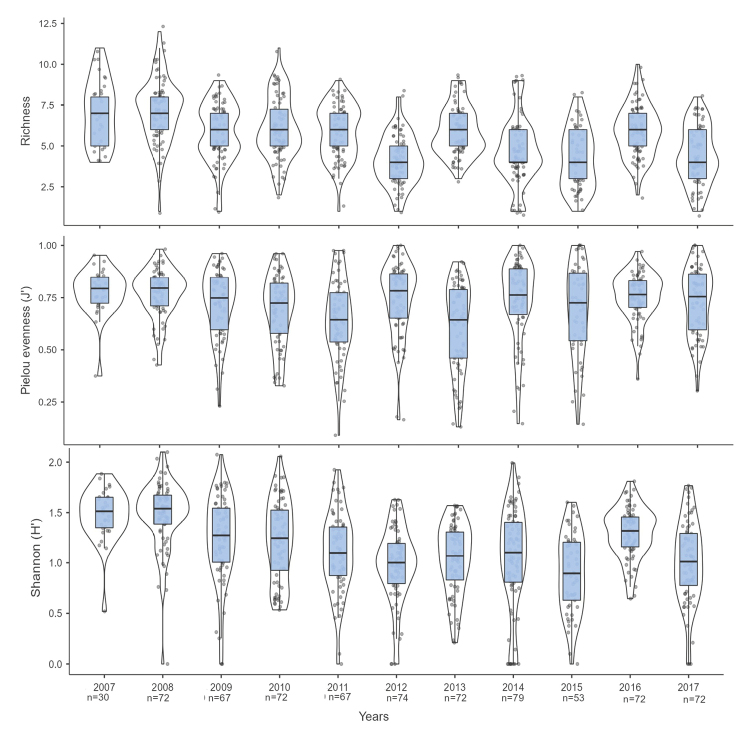
Ecological indicators of invertebrate species richness (S), Shannon-Wiener diversity (H’), and Pielou evenness (J’) by transect. The inferior and superior sides of each blue rectangle represent the first and third quartiles (P25 and P75), respectively, and the median is represented by the horizontal black line. The points indicate the values of each data point, while the line surrounding each box plot shows the probability density.

In the ISPMBR, the average invertebrate richness was 5.50 ± 2.02 species/transect (mean ± SD). The year with the highest richness was 2008 (7.19 ± 1.93 species/transect), while the year with the lowest richness was 2012 (4.12 ± 1.50 species/transect; Fig. 3). The mean H’ value was 1.15 ± 0.42, and the highest H’ values were observed in 2007 (1.48 ± 0.27) and 2008 (1.48 ± 0.33), while the lowest H’ values ​​were recorded in 2012 (0.98 ± 0.37) and 2015 (0.90 ± 0.39; Fig. 3). The average J’ value was 0.65 ± 0.18, and the highest J’ values were observed in 2012 (0.72 ± 0.17) and 2014 (0.72 ± 0.19), while the lowest J’ value was found in 2013 (0.53 ± 0.19; Fig. 3). The Kruskal-Wallis analysis of the richness, diversity, and evenness indicators indicated significant differences among years (p < 0.001; Suppl. material 4: Table S4).

**Figure 4.**
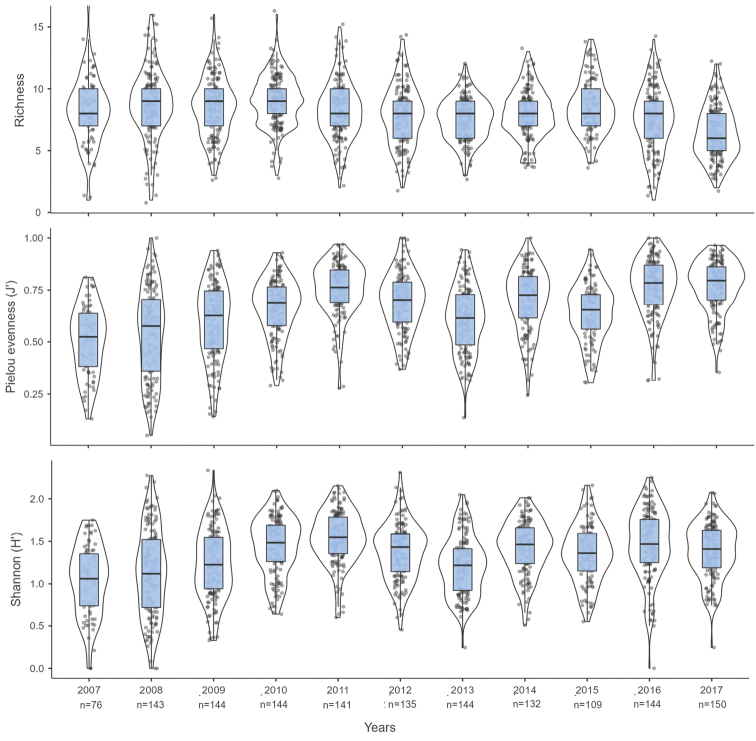
Ecological indicators of fish species richness (S), Shannon-Wiener diversity (H‘), and Pielou evenness (J’) by transect. The inferior and superior sides of each blue rectangle represent the first and third quartiles (P25 and P75), respectively, and the median is represented by the horizontal black line. The points indicate the values of each data point, while the line surrounding each box plot shows the probability density.

In the case of fish, the average S value (mean ± SD) was 8.02 ± 2.43 species/transect. The highest S value was observed in 2010 (9.05 ± 2.09 species/transect), while the lowest S value was observed in 2017 (6.43 ± 2.13 species/transect; Fig. 4). The mean H’ value was 1.34 ± 0.40, and the highest H’ values were observed in 2011 (1.54 ± 0.31), 2016 (1.46 ± 0.40), and 2010 (1.44 ± 0.34), while the years with the lowest H’ values were 2007 (1.03 ± 0.41) and 2008 (1.12 ± 0.51; Fig. 4). The average J’ value was 0.54 ± 0.19, and the highest and lowest J’ values were found in 2011 (0.62 ± 0.16) and 2007 (0.39 ± 0.16), respectively (Fig. 4). The Kruskal-Wallis analysis of richness, diversity, and evenness indicators indicated significant differences among years (p < 0.001; Suppl. material 4: Table S4).

**Figure 5.**
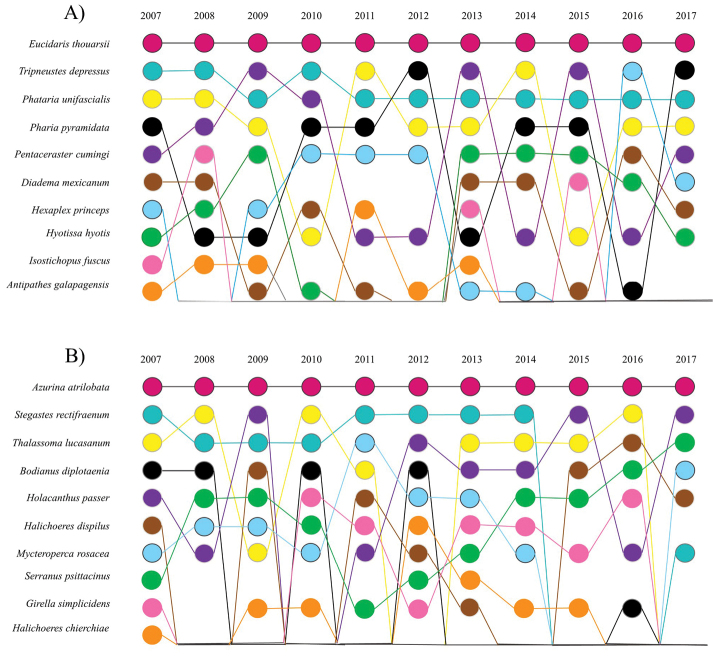
Yearly ranks of the ten most abundant species of invertebrates **A** and fishes **B**.

When yearly changes in the rank abundance curves (Table 5) were assessed, we observed that differences in beta diversity (changes in richness, species evenness, species gains/losses, and curve change) between years was low change for invertebrate and fishes. Additionally, we observed that the ranks of the ten most abundant species for invertebrates and fishes were similar over the last 10 years, where *Eucidaristhouarsi* and *Azurinaatrilobata* were the species with higher abundance during the sampling widow (Figure 5).

**Table 5.** Differences in the alpha diversity metrics of invertebrates and fishes between 2007 and 2017.

Invertebrates		Change in S	Curve change	Change in evenness	Species loss	Species gains
2007	2008	0.214	0.107	-0.006	0.000	0.214
2008	2009	-0.032	0.134	-0.022	0.129	0.097
2009	2010	-0.100	0.104	-0.002	0.200	0.100
2010	2011	0.036	0.115	-0.020	0.107	0.143
2011	2012	-0.034	0.138	0.068	0.172	0.138
2012	2013	-0.120	0.157	-0.045	0.160	0.040
2013	2014	0.120	0.094	0.008	0.040	0.160
2014	2015	-0.036	0.102	0.032	0.179	0.143
2015	2016	0.036	0.138	-0.032	0.143	0.179
2016	2017	-0.250	0.175	0.027	0.250	0.000
**Fishes**		**Change in S**	**Curve change**	**Change in evenness**	**Species loss**	**Species gains**
2007	2008	0.000	0.143	-0.008	0.154	0.154
2008	2009	0.109	0.108	0.012	0.091	0.200
2009	2010	-0.073	0.093	-0.006	0.164	0.091
2010	2011	0.019	0.146	0.035	0.113	0.132
2011	2012	-0.019	0.129	-0.026	0.132	0.113
2012	2013	-0.040	0.124	-0.016	0.120	0.080
2013	2014	-0.149	0.123	0.035	0.213	0.064
2014	2015	0.048	0.126	-0.027	0.071	0.119
2015	2016	0.196	0.116	0.015	0.039	0.235
2016	2017	-0.017	0.137	-0.016	0.172	0.155

## Discussion

The distribution of a species is determined by both abiotic and biotic conditions, in addition to the accessibility of areas based on the dispersal limits of the species and the region in which it originally evolved. Abiotic and biotic conditions vary greatly among the different regions of the Gulf of California, and a south-north diversity gradient has been identified. This gradient has been reported by Thompson (1979), Brusca et al. (2005), Fernández et al. (2018), and Olivier et al. (2018) and is given by the differences among fauna, flora, and oceanographic characteristics in the Gulf of California. The southern region contains tropical species and warm waters, while the northern region (where the ISPMBR is found) is characterized by tropical, subtropical, and temperate fauna; colder waters; and high chlorophyll concentrations and primary production (Escalante et al. 2013). Not surprisingly, the central region is a transition zone between the northern and southern regions. Despite these characteristics, most of the Gulf of California fauna have been reported to have tropical affinity and derive from the eastern Pacific (Brusca 2010), which agrees with what was found in this study. A total of 45.71% and 64.38% of the invertebrate and fish species recorded in the ISPMBR, respectively, are widely distributed in the eastern Pacific.

In the ISPMBR, a total of 11 protected species (i.e., three invertebrate and eight fish species) were identified, which constitute 10% of all species surveyed in this study. The clarion angelfish (*H.clarionensis*) was the only species recorded in only one year (i.e., 2009). The presence of tropical species has been recorded in several studies of the Gulf of California (Gónzalez-Cuellar et al. 2013; Martínez-Torres et al. 2014; Fernández-Rivera Melo et al. 2015; Gonzalez-Acosta et al. 2016; Fernández-Rivera Melo et al. 2018; Reyes-Bonilla et al. 2019). These studies have indicated that the distribution ranges of fish species have expanded, possibly due to climate change given the increase in water temperature that has been mainly reported for the central region of the Gulf of California (García-Morales et al. 2017; Robles-Tamayo et al. 2018). The presence of the clarion angelfish may have been due to a moderate El Niño during 2009 (https://ggweather.com/enso/oni.htm) and to the resulting expansion of the distribution of this tropical species into temperate waters (Hernández-Velasco et al. 2016). However, we assumed that this specimen either moved to another area in the Gulf of California with more tropical characteristics or died without being able to colonize the site.

Two species (*S.limbatus* and *I.fuscus*) showed decreases in abundance over the course of this study, which may have been due to commercial harvest (Cudney-Bueno and Rowell 2008; Calderón 2019; Villalejo-fuerte et al. 2020). From 2012–2017, *P.mazatlanica*, *M.jordani*, and *H.passer*, which are also commercial species, presented relatively stable abundance values. This may be explained by the comparatively low prices of *P.mazatlanica* (US$ 1/kg), *M.jordani* (US$ 3/kg), and *H.passer* (US$ 4/ind.) compared to those of *S.limbatus* (US$ 14/kg) and *I.fuscus* (US$ 30/kg), which cannot sufficiently compensate for the amount of time needed to reach the island during each fishing (ca. three hours). It may also be that fishers in the region do not have markets for *P.mazatlanica*, *M.jordani*, and *H.passer*.

The rocky reefs of the ISPMBR are essential for fishing activities. In this study, 64% of the species recorded were of commercial importance in the region. Monitoring the abundances and average sizes of these species is crucial to ensuring the sustainable use and conservation of this marine protected area (Dames et al. 2020). In this sense, the leopard grouper (*M.rosacea*) and yellow snapper (*L.argentiventris*) showed relatively stable abundance values and average sizes that increased over the 10 years of this study, which was probably due to three principal factors. First, these species are subject to relatively low fishing pressure. The natural protected area was decreed in 2002 and includes a third of the island coastline with 3.7% of the total area declared a no-fishing zone. The recovery of the abundance and sizes of commercially important species due to the establishment of natural protected areas has been documented in diverse marine regions (Aburto-Oropeza et al. 2011; Chirico et al. 2017). Second, a sufficient degree of connectivity among the islands and coasts in the region appears to be present. Connectivity has important implications for the persistence of metapopulations and the ability of individual populations to recover from disturbance (Green et al. 2015). Marinone (2012) conducted a connectivity study by modeling the movement of particles in the Gulf of California and found that the ISPMBR receives larvae from the coasts of Sonora, Sinaloa, and the MIR. Finally, natural larval retention and recruitment also play a role in population recovery. Soria et al. (2014) conducted a study in the ISPMBR on larval retention and found that a certain degree of retention occurs during May, which would indicate that the natural marine area has also helped to protect these commercially important species, both within the reserve and in the larger MIR.

Studies of invertebrates in the Gulf of California have focused on generating lists of both conspicuous and cryptic species of various invertebrate taxa (e.g., cnidarians, echinoderms, mollusks, and crustaceans) in the northern, central, and southern regions (Brusca et al. 2005; Brusca 2007), while other studies have primarily focused on descriptions of the richness and diversity of echinoderms (e.g., starfish, urchins, and sea cucumbers). In Loreto, Holguín Quiñoez et al. (2000) recorded 26 species of echinoderms, while Luna-Salguero and Reyes-Bonilla (2010) reported nine species of starfish. In Bahía de los Ángeles, Herrero-Perézrul et al. (2008) recorded 11 species of starfish and urchins. The species richness of the ISPMBR reported in this study (16 species; seven stars, eight urchins, and one sea cucumber) is very similar to what has been reported in the protected areas of Loreto and Bahía de los Ángeles, which may be due to the proximity of the three areas or to the evaluation of these invertebrates in shallow, rocky reefs. The specific richness of this study was less than that found by Holguín Quiñonez et al. (2000), although those authors sampled muddy and sandy substrates as well as rocky reefs.

The studies that have been published on ichthyofauna in the MIR are scarce. Del Moral-Flores et al. (2013) conducted a review of bibliographic sources and collections, reporting 36 species of fish in the ISPMBR. Mascareñas-Osorio et al. (2011) and Fernández-Rivera Melo et al. (2018) carried out visual surveys in Bahía de los Ángeles and found 70 and 34 species of fishes, respectively. Finally, the survey results reported by the management program of the ISPMBR (CONANP 2007) include a total of 84 species of elasmobranchs and bony fish (both conspicuous and cryptic) associated with different habitats (i.e., sandy bottoms, rocky reefs, and *Sargassum* spp. forest). The number of species reported in this study (73) agrees with the number of species reported by both CONANP (2007) and Mascareñas-Osorio et al. (2011). Considering the number of invertebrate and fish species recorded in this study, the number of invertebrates and fish species found in the ISMPRB increases to 45 and 101, respectively. Also, the clarion angelfish was registered for the first time in MIR (GBIF 2021).

The most abundant invertebrate species in this study (*E. thouarsi*, *P.unifascialis*, and *P.pyramidata*) are also species that have been reported to be abundant in the Gulf of California (Luna-Salguero and Reyes-Bonilla 2010; Reyes-Bonilla et al. 2005; Herrero-Perézrul et al. 2008; Suppl. material 2: Table S2). The three species of fish with the highest abundance (*A.atrilobata*, *S.rectifraenum*, and *T.lucasanum*; Suppl. material 3: Table S3) were the same as those reported in other studies of the Gulf of California (Pérez-España et al. 1996; Sánchez- Ortiz et al. 1997; Thompson et al. 2000; Aburto-Oropeza and Balart 2001; Viesca-Lobatón et al. 2008; Fernández-Rivera Melo et al. 2018). The fact that the most abundant species have remained the same for more than 10 years indicates that no changes have severely impacted the structure or function of the invertebrate or fish communities present (Gray 1989; Day et al. 2018).

As Nybakken (1997) and Odum (1983) mention, communities are not static units in time; their structure and composition change to varying degrees during cyclical periods of varying durations. During these periods, the presence and dominance of species also changes, giving rise to sequences that can be more ordered, less ordered, or random. Ecological indicators allow for an evaluation of changes in community structure and function over time in the face of natural stressors in the form of biotic factors (e.g., food availability, competition, and predation), abiotic factors (e.g., temperature, salinity, currents, and pH), and anthropogenic stress (e.g., sedimentation, eutrophication, pollution, and overfishing). In the case of the ISPMBR, ecological indicators will help in evaluations of the conservation status of the protected area, taking into account the objectives described in the management program (e.g., maintaining biodiversity and ecological processes).

The H’ and J’ indices are useful for monitoring the conservation status of ecosystems because they consider the total number of species and the homogeneity with which their abundances are distributed. Both components of community structure have been interpreted against a background of important ecological processes (Magurran 2003). It may be deduced that a complex community with a greater number of interactions and stability is present when species diversity is high compared to that when species diversity is low (McCann 2000; Jorgensen and Muller 2000; Magurran 2003). These considerations have led to diversity indices being used to reflect the conservation status of ecosystems, assuming that the conservation status improves as diversity increases, regardless of how it is measured (UNESCO 2003; Mac Nally and Fleishman 2004; Spellerberg 2005).

The diversity and evenness results allowed us to identify trends for both the invertebrate and fish communities in this study. Between 2007 and 2011, a decrease in invertebrate diversity was observed, while a gradual increase in fish diversity was recorded (Fig. 3 and Fig. 4). This result may be related to the abundance distributions. The invertebrate group contained three species whose abundances amounted to more than 50% of the total and gradually increased between 2007 and 2011. In contrast, the fish group showed relatively homogeneous abundances as the years passed. In addition, the two communities did not present significant changes in diversity between 2012 and 2017, which might indicate stability within the two communities.

Disturbance plays a central role in structuring communities, and the prevalence of human-induced disturbance has resulted in wide-ranging effects on biodiversity and ecosystem functioning and species abundance in particular (Matthews and Whittaker 2014). The stability of the ecological indicators evaluated in the ISPMBR may be explained by the presence of low-level anthropogenic stressors and the resilience of the invertebrate and fish communities. Although this island is located in an area with abiotic stressors of medium to high intensity, the complexity of the habitats and substrates present (e.g., shallow and deep rocky reefs, mangroves, walls, and brown algae) provides protection for the different species and life stages present (Aburto-Oropeza and Balart 2001; Dominic-Arosemena and Wolff 2006). In addition, individuals of many species move vertically in the water column to protect themselves from storms and increases in sea surface temperature (Currey et al. 2015). Furthermore, high fidelity has been observed in the species present, and their abundances have remained relatively stable over time (Table 5, Fig. 5). Moreover, the fidelity of species, such as parrotfishes, surgeonfishes, goat fishes, snappers, and groupers, to specific geographic areas has been reported in the Gulf of California, Catalina Island (California) and Hawaii (Tinhan et al. 2014; Topping et al. 2006; Meyer et al. 2010). Given these conditions, it can be assumed that the island has shown high resilience from 2007 to 2017. However, further assessments of the community structure of the ISPMBR are needed due to the observed increase in illegal fishing that has resulted from the closures of the northernmost fishing areas in 2019 and to the impacts of synoptic scales (e.g., climate change, El Niño, La Niña, and seasonal cycles) and mesoscale (e.g., the thermocline, surface circulation, gyres, storms, and upwelling) processes in the region that could increase in frequency, duration, or intensity in the future (Páez-Osuna et al. 2016).

The results of this study constitute the first analysis of the community structure of the ISPMBR, with emphasis on the distribution, conservation, and use of the invertebrates and fish species present in the shallow rocky reefs of the natural protected area. A taxonomic list based on an 11-year data set is also presented.

## Conclusions

In this study, we analyzed eleven years of survey data of the most oceanic marine protected area in the Gulf of California: the ISPMBR. We observed that invertebrate and fish fauna in the rocky coastal reefs present stability with regard to the ecological indicators considered in this study. The marine protected area is both ecologically and commercially important. A total of 108 species were recorded (35 invertebrates and 73 fishes), of which 54 are commercially important (12 invertebrate and 42 fish species). Two principal trends were observed. First, the abundance of commercially important invertebrate species is decreasing, which is probably due to their high monetary value and illegal fishing (e.g., the sea cucumber *I.foscus*). Second, commercially important fish species maintained their abundance overall, albeit with periods of increase. The use of long-term monitoring data can provide a more realistic picture of the dynamics inside a marine protected area, which may then be used to evaluate its performance.

## Supplementary materials

Supplementary material 1 Table S1 Data type Xlsx file. Explanation note Average (standard deviation) for ecological indicators of invertebrates and fishes, species richness (S), Shannon-Wiener diversity (H’), and Pielou evenness (J’). https://binary.pensoft.net/file/599480This dataset is made available under the Open Database License (http://opendatacommons.org/licenses/odbl/1.0/). The Open Database License (ODbL) is a license agreement intended to allow users to freely share, modify, and use this Dataset while maintaining this same freedom for others, provided that the original source and author(s) are credited.Amador-Castro Imelda G., Fernández-Rivera Melo Francisco J., Torre, Jorge

Supplementary material 2 Table S2 Data type Xlsx file. Explanation note Total abundance of invertebrates. Abbreviations: n = number of transects. https://binary.pensoft.net/file/599481This dataset is made available under the Open Database License (http://opendatacommons.org/licenses/odbl/1.0/). The Open Database License (ODbL) is a license agreement intended to allow users to freely share, modify, and use this Dataset while maintaining this same freedom for others, provided that the original source and author(s) are credited.Amador-Castro Imelda G., Fernández-Rivera Melo Francisco J., Torre, Jorge

Supplementary material 3 Table S3 Data type Xlsx file. Explanation note Total abundance of fishes. Abbreviations: n = number of transects. https://binary.pensoft.net/file/599482This dataset is made available under the Open Database License (http://opendatacommons.org/licenses/odbl/1.0/). The Open Database License (ODbL) is a license agreement intended to allow users to freely share, modify, and use this Dataset while maintaining this same freedom for others, provided that the original source and author(s) are credited.Amador-Castro Imelda G., Fernández-Rivera Melo Francisco J., Torre, Jorge

Supplementary material 4 Table S4 Data type Xlsx file. Explanation note Results of the post-hoc analysis for ecological indicators of invertebrates and fishes, species richness (S), Shannon-Wiener diversity (H’), and Pielou evenness (J’). https://binary.pensoft.net/file/599483This dataset is made available under the Open Database License (http://opendatacommons.org/licenses/odbl/1.0/). The Open Database License (ODbL) is a license agreement intended to allow users to freely share, modify, and use this Dataset while maintaining this same freedom for others, provided that the original source and author(s) are credited.Amador-Castro Imelda G., Fernández-Rivera Melo Francisco J., Torre, Jorge
